# Genetic Polymorphism of Epidermal Growth Factor Gene as a Predictor of Hepatocellular Carcinoma in Hepatitis C Cirrhotic Patients 

**DOI:** 10.31557/APJCP.2020.21.7.2047

**Published:** 2020-07

**Authors:** Ibrahim Baghdadi, Khaled Abu Ella, Ahmed El Shaarawy, Elsayed Elshayb, Hala S El-Rebey, Mohamed M El Hoseeny, Mary Naguib, Ali Nada

**Affiliations:** 1 *Internal Medicine Department, Faculty of Medicine, Menoufia University, Menoufia, Egypt. *; 2 *Liver and Hepatobiliary surgery Department, National Liver Institute, Menoufia University, Menoufia, Egypt. *; 3 *Clinical Pathology Department, National Liver Institute, Menoufia University, Menoufia, Egypt. *; 4 *Pathology Department, Faculty of Medicine, Menoufia University, Menoufia, Egypt. *; 5 *Hepatology Department, Mahlla teaching liver hospital, El Mahlla, Gharbeya, Egypt. *; 6 *Hepatology Gastroenterology Department, National Liver Institute, Menofia University. Egypt. *

**Keywords:** Epidermal growth factor gene polymorphism, Hepatocellular Carcinoma, Hepatitis C Virus

## Abstract

**Background:**

In Egypt, the incidence of hepatocellular carcinoma (HCC) is approximately 4.7% of chronic liver disease patients due to (HCV) infection. Epidermal growth factor (EGF) plays an important role in hepatocyte regeneration. A functional polymorphism in *EGF 61A>G* was identified; itwas associated with higher risk of HCC.

**Objectives:**

to investigate the correlation between the epidermal growth factor (*EGF*) polymorphism and the risk of hepatocellular carcinoma (HCC) in hepatitis C viral (HCV) cirrhotic patients as well as its relation to EGF protein expression in HCC tissue.

**Patients and methods:**

this casecontrol study was conducted on 75 HCV cirrhotic patients including 50 HCC patients (25 withresectable HCC and 25 with advanced unresectable HCC) and 25 healthy persons were included. EGF genotype was detected by restriction fragment length polymorphism. EGF expression in HCC tissue biopsiesfrom patientswhounderwent surgical resection was done by immunohistochemical examination.

**Results:**

The GG genotype was associated with significant increased risk of HCC compared to AA genotypes (P=0.031) in cirrhotic group. The G allele had a highly significant risk of HCC compared to allele Ain recessive model GG vs. AG+AA (P=0.036) rather than in the dominant model GG +AG vs. AA (P=0.66). There was significant increased expression of EGF in tumour tissues in patients with GG genotype compared to AG genotype and AA genotype p= 0.019.

**Conclusion:**

*EGF *gene polymorphism (GG genotype) had a significant risk of HCC development in cirrhotic patients. This is confirmed by increased *EGF* expression in liver tumor tissue from HCC patients.

## Introduction

Hepatocellular carcinoma (HCC) is the fifth most common cancer and the third leading cause of cancer deaths worldwide (Thrift et al., 2017). Risk factors vary widely in different geographic regions worldwide (Lavanchy and Kane, 2016). In Egypt hepatitis C virus (HCV) infection is a main cause as Egypt has high prevalence of HCV infection where viremia was reported as 7.3% (Waked et al., 2014).

Diagnosis of HCC patients occur at the late stage, allowing only minority of the patients to be candidates for possible curative treatments (Li et al., 2010). Pathogenesis of HCC usually correlates with the presence of continuous inflammation and hepatocyte regeneration associated with chronic hepatitis and hepatic cirrhosis(Ringelhan et al., 2018). Genetic factors also have an important role in HCC pathogenesis (Yuan et al., 2013).

Therefore, studying different biomarkers associated with the increased risk of HCC would allow better screening of highrisk populations for HCC and help to improve prevention and treatment (Li et al., 2010).

Epidermal growth factor (EGF) plays a significant role in cell proliferation, differentiation and tumorigenesis of epithelial tissues (Zhong et al., 2012).The *EGF 61A>G* polymorphism (*rs4444903*) is a functional SNP in the 5’ untranslated region of the *EGF* gene (Xu et al, 2010, Zhang et al., 2010). It results in higher EGF levels in individuals with EGF genotype G/G in comparison to the A/A genotype (Almeida et al., 2010). 

Previous studies have shown that *EGF rs4444903 SNP* could result in increased risk of tumorigenesis in HCC (Zhong et al., 2012). However, other studies have indicated that thereis no significant association (Qi et al., 2009).Thus, we aimed to detect the correlation between *EGF* gene polymorphism and risk of HCC in Egyptian HCVcirrhotic patients. Also validate EGFprotein expression in HCC tissue related to this polymorphism.

## Materials and Methods

*Patients and methods*

*Study population*

This case-control study included 75 patients and 25 healthy individuals matched in age and sex as a control group. Patients were recruited from HCC Clinic, Hepatology Unit,National Liver Institute, Menoufiya University in the duration between February 2017 and February 2018. Study was conducted according to the Declaration of Helsinki. All participants provided written informed consent, and the Ethics Committee of National Liver Institute, Menoufiya University approved the study protocol.

Adult cirrhotic HCV patients (> 18 years) were eligible to the study. Diagnosis of cirrhosis was done by clinical evaluation, laboratory investigations and abdominal ultrasonography (US). Patients wereclassified according toabdominal US, abdominal tri-phasic computed tomography (CT) and serum alpha fetoprotein (AFP) level into HCC patients and cirrhotic patients with no evidence of HCC.

HCC patients were grouped to patients with surgicalresectable HCC and patients with advanced unresectable HCC (Multicentric hepatic focal lesions with and/ or portal vein thrombosis). Healthy persons, age and sex matched, were enrolledas control group (they were clinicallyfree with normal laboratory investigations, normal abdominal ultrasonography, and no history of liver disease).

Patients withhereditary hepatic diseases,autoimmune liver disorders, other liver cancers,liver disease other than HCV andhistory of radiological intervention for management of HCC were excluded. 

*Laboratory investigations*

*Routine laboratory investigations*

Liver, renal function testsand random blood sugar were performed on Cobas- 6000 auto analyser (Roche diagnostics- GmbH, D-68305 Mannheim, Germany), prothrombin concentration andinternational normalized ratio (INR) on BFT II Analyzer (Dade Behring Marburg GmbH, D-35041 Marburg, Germany) and seum α-fetoprotein level on Cobas e411 immunoassay analyser (Roche diagnostics- GmbH, D-68305 Mannheim, Germany). 

*Specific investigations*

I) DNA extraction and EGF genotyping:

Genomic DNA was extracted from venous blood sample using Zymo Quick-gDNA™ MiniPrep DNA Purification Kit (Zymo Research, CA, USA). The *EGF 61A > G* Polymorphism (*rs4444903*) was detected using polymerase chain reaction andrestriction fragment length polymorphism (PCR- RFLP) as previously described (Amend et al., 2004; Suenaga et al., 2013).

PCR amplification of EGF was performed using 1μL of each of the following primers Forward: 5′-TGTCACTAAAGGAAAGGAGGT-3′ and reverse 5′-TTCACAGAGTTTAACAGCCC-3′inthe following reaction mixture: 12.5 μl of MyTaq™ Red Mix master mix (Bioline, MA, USA), 5.5 μl of nuclease-free Water and 5 μl of extracted DNA. Amplification occurred through the following conditions: initial denaturation at 95°C for 5 minutes, followed by 35 cycles; 95°C for 45 seconds, 51°C for 45 seconds, 72°C for 45 minute and final extension step of 10 minutes at 72°C using Perkin Elmer Gene Amp PCR System 2400 Thermal Cycler.The successful amplification of a 242 bp region of the* EGF 61A > G* Polymorphism was confirmed using 3% agarose gel electrophoresis. 

Then 10 μL DNA amplification product was digested with 1 μL Fast Digest AluI restriction enzyme (New England Biolabs) for 5-15 min at 37°C. Digestion of the 61*G allele produced 15, 34, and 193 bp fragments, while digestion of the 61*A allele produced 15, 34, 91, and 102 bp fragments.

*Detection of EGF in HCC tissue of patients underwent liver resection*

Two biopsies were taken from each patient in resectable HCC group, one from neoplasticlivertissue and another one from adjacent non- neoplastic tissue. Both stained with hematoxylin andeosin (H and E) were examined under the light microscope to confirm the diagnosis of HCC of the neoplastic liver tissue and cirrhosis of the adjacent non-neoplastic tissue.

Then stained immunohistochemically for EGF expression by streptavidin-biotin amplified System.EGF expression was considered positive when > 5% of the cells showed cytoplasmic brown staining. H score was applied to evaluate the studied cases according to (Bilalovic et al., 2004), where both intensity (scored 1-3 as 1= mild, 2=moderate and 3=strong) and percentage of positive cells were considered.

The intensity score is multiplied by the percentage of cells which stain with each level of intensity, and the sum of these mathematically products is expressed as H score. 

H score formula= strong intensity (3) x percentage + moderate intensity (2) x percentage+ mild intensity (1) x percentage.

*Statistical analysis*

Results were statistically analyzed by using statistical package of social sciences (SPSS 22.0, IBM/SPSS Inc., Chicago, IL). Categorical data were presented as number and percentage while quantitative data were expressed as mean and standard deviation. Comparison of continuous data between more than two groups was made by using one way ANOVA for parametric data and Kruskal-Wallis test for nonparametric data with post-tests (Turkey and Dunn test, respectively). Chi square test was used for comparison between Categorical data. P-value < 0.05 was considered significant.

## Results

*Characteristics of the studied subjects*

This study included 75 HCV related cirrhotic patients;25 (33.3%) patients with surgical resectable HCC, 25 (33.3%) patients with advanced unresectable HCC and 25 (33.3%) cirrhotic HCV patients with no evidence of HCC, also 25 subjects were enrolled as control group.HCC, cirrhotic patients and controls had similarage and gender distribution.They were mostly male (82%, 72% and 64% respectively, p= 0.219) (Table 1).

We couldn’t detect significant difference between HCC group and cirrhotic patients group regarding liver and kidney function tests. However, AFP was significantly higher in HCC group compared to cirrhotic patient group. On the other hand,HCC patients showed significantly elevated ALT, AST, total bilirubin, INR, urea levels and significantly decreased level of albumin compared to control group (Table 2).

*Frequency of EGF 61A>G SNP among studied groups and its risk effect*

G allele showed statistical higher frequency in HCC group (63 %) compared to cirrhotic patients (42%) and control group (38%) (p= 0.005), with increased GG genotype in HCC group (40%) compared to cirrhotic patient group (16%) and control group (12%) (p= 0.029). 

Compared to cirrhotic patients,GG genotype was associated with significant increased risk of HCC compared to AA genotype with OR (95% CI) 5.71, (P=0.031). The G allele carried a highly significant risk of HCC compared with allele A OR (95% CI) 2.35, (P value =0.015). The variant G allele showed a significant association with HCC risk in the recessive model GG vs. AG+AA (P=0.036) rather than the dominant model GG +AG vs. AA (P=0.066)(Table 3). We couldn’tdetect significant difference in genotype distribution and allele frequencies of EGF polymorphism between resectable and unresectable HCC patients (Table 4) 

*Studying effect of genotype distribution on resectable HCC group*

There was similarity in age and gender distribution regarding different genotypes in resectable HCC group. We couldn’t detect significant difference infoci size or AFP levels. However, there was significant increased expression of EGF in tumour tissues (200.00 ± 28.78) in patients with GG genotype compared to AG genotype (162.31 ± 30.86) and AA genotype (152.50 ± 35.00), p= 0.019. Also, expression of EGF in surrounding cirrhotic tissue was elevated in GG genotype (162.50 ± 53.12) compared to AG genotype (138.46 ± 53.52) and AA genotype (140.00 ± 73.48), however we couldn’t find significant difference, p= 0.626 (Table 5).

**Table 1:** Statistical Analysis of Demographical Data in HCC, Cirrhosis and Control Groups

Parameters	HCC (n = 50)	Cirrhosis (n = 25)	Control (n = 25)	Significance test	*P*-value
Age (year)				F = 0.70	0.498 ^NS, a^
Mean ± SD	57.44 ± 7.41	58.12 ± 6.16	55.76 ± 8.28		
Range (min-max)	43 – 76	45 – 71	37 - 71		
Gender [n (%)]				*χ* ^2^ =3.04	0.219 ^NS, b^
Male	41 (82.0)	18 (72.0)	16 (64.0)		
Female	9 (18.0)	7 (28.0)	9 (36.0)		

%, percent within group; ^a^, ANOVA test; b, Pearson chi-square test; NS, Non significant at P-value ≥ 0.05; SD, Standard deviation; n, numberof patients; HCC, Hepatocellular carcinoma; GI, group1; Min, Minimum,; Max, Maximum

**Table 2:** Statistical Analysis Ofbiochemical Lab Parameters in HCC, Cirrhosis and Control Groups

Biochemical parameters	HCC	Cirrhosis	Control	Significance test	Pairwise comparisons*
	(n = 50)	(n = 25)	(n = 25)		
ALT (U/L)				*χ* ^2^= 41.37	*P*_1_=1.000 ^NS^
Median (IQR)	63.50 (44.75)	56.00 (23.50)	26.00 (11.00)	*P*-value	*P*_2_< 0.001^HS^
Range (min-max)	11.00 - 404.00	31.00 - 98.00	11.00 - 51.00	< 0.001^HS,a^	*P*_3_< 0.001^HS^
AST (U/L)				*χ* ^2^= 42.17	*P*_1_=1.000^NS^
Median (IQR)	67.00 (68.25)	65.00 (37.50)	24.00 (12.50)	*P*-value	*P*_2_< 0.001^HS^
Range (min-max)	12.00 - 534.00	23.00 - 243.00	12.00 - 49.00	< 0.001^HS,a^	*P*_3_< 0.001^HS^
Total bilirubin (mg/dL)				*χ* ^2^= 34.79	*P*_1_=0.213 ^NS^
Median (IQR)	1.65 (2.90)	2.10 (1.45)	0.80 (0.20)	*P*-value	*P*_2_< 0.001^HS^
Range (min-max)	0.30 - 10.00	1.30 - 5.20	0.50 - 1.30	< 0.001^HS,a^	*P*_3_< 0.001^HS^
Albumin (g/dL)				*χ* ^2^= 56.69	*P*_1_= 0.001^HS^
Median (IQR)	3.30 (1.20)	2.60 (0.84)	4.30 (0.60)	*P*-value	*P*_2_< 0.001^HS^
Range (min-max)	1.90 - 4.60	1.70 - 3.30	3.70 - 5.00	< 0.001^HS,a^	*P*_3_< 0.001^HS^
INR value				*χ* ^2^=28.67	*P*_1_=0.153^NS^
Median (IQR)	1.40 (0.60)	1.90 (0.70)	1.00 (0.20)	*P*-value	*P*_2_< 0.001^HS^
Range (min–max)	0.90 - 3.10	1.00 - 3.10	0.90 - 1.30	< 0.001^HS,a^	*P*_3_< 0.001^HS^
Urea (mg/dL)				*χ* ^2^ = 22.98	*P*_1_=1.000^NS^
Median (IQR)	54.00 (22.00)	54.00 (44.50)	33.00 (12.50)	*P*-value	*P*_2_< 0.001^HS^
Range (min–max)	21.00 - 190.00	12.00 - 98.00	17.00 - 53.00	< 0.001^HS,a^	*P*_3_= 0.001^HS^
Creatinine (mg/dL)				*χ* ^2^ = 2.25	–
Median (IQR)	1.00 (0.53)	1.10 (0.70)	1.00 (0.20)	*P*-value	
Range (min–max)	0.40 - 2.30	0.40 - 2.40	0.70 - 1.30	= 0.325 ^NS,a^	
RBS (mg/dL)				*χ* ^2^ = 5.40	–
Median (IQR)	110.00 (47.25)	98.00 (44.00)	105.00 (27.00)	*P*-value	
Range (min–max)	65.00 - 364.00	68.00 - 243.00	62.00 - 145.00	=0.067 ^NS,a^	
AFP (ng/mL)				*z* = 4.58	–
Median (IQR)	62.50 (226.25)	17.00 (16.50)	–	*P*-value	
Range (min–max)	5.40 - 151000.00	3.10 - 68.00	–	< 0.001^HS,b^	

IQR, Interquartile range; NS, Non significant at P-value ≥ 0.05; HS, Highly significant at P-value < 0.01; ^a^, Kruskal-Wallis test; ^b^, Mann-Whitney U test ; *, Multiple pairwise comparisons adjusted by Bonferronipost hoc test; *P*_1_, P-value for the difference between HCC and cirrhosis groups; *P*_2_, P-value for the difference between HCC and control groups; *P*_3_, P-value for the difference between cirrhosis and control groups.

**Table 3:** Comparison of Genotypes Distribution and Allele Frequencies of EGF Polymorphism (61 A/G) in HCC versus Cirrhoticpatients

EGF Polymorphism 61 A/G	HCC (n = 50)	Cirrhosis (n = 25)	OR (95% CI)	*P*-value
Genotypes [n (%)]				
GG	20 (40.0)	4 (16.0)	5.71 (1.30 - 25.03)	0.031^S, a^
AG	23 (46.0)	13 (52.0)	2.02 (0.60 - 6.86)	0.255N^S, b^
AA	7 (14.0)	8 (32.0)	Ref.	–
Dominant model^1^				
GG +AG	43 (86.0)	17 (68.0)	2.89 (0.91 - 9.22)	0.066^NS, a^
AA	7 (14.0)	8 (32.0)	Ref.	–
Recessive model^2^				
GG	20 (40.0)	4 (16.0)	3.50 (1.04 - 11.73)	0.036^S, b^
AG+AA	30 (60.0)	21 (84.0)	Ref.	–
Alleles [n (%)]				
G	63 (63.0)	21 (42.0)	2.35 (1.18 - 4.70)	0.015^S, b^
A	37 (37.0)	29 (58.0)	Ref.	–

^a^, Fisher’s Exact test; ^b^, Pearson Chi-Square test; %, percent of genotype or allele within group-NS : Non significant at P-value ≥ 0.05; ^1^, Dominant model, (homozygous type + hybrid type)vs. wild type; ^2^, Recessive model,homozygous vs. (hybrid type+ wild type)

**Table 4:** Comparison of Genotype Distribution and Allele Frequencies of EGF Polymorphism (61 A/G) Inresectable versus Unresectable HCC Patients

EGF Polymorphism 61 A/G	Resectable HCC (n = 25)	Unresectable (n = 25)	OR (95% CI)	*P*-value
Genotypes [n (%)]				
GG	8 (32.0)	12 (48.0)	2.00 (0.35 - 11.44)	0.662 ^NS, a^
AG	13 (52.0)	10 (40.0)	1.03 (0.19 - 5.66)	1.000 ^NS, a^
AA	4 (16.0)	3 (12.0)	Ref.	–
Dominant model^1^				
GG +AG	21 (84.0)	22 (88.0)	1.40 (0.28 – 7.00)	1.000 ^NS, a^
AA	4 (16.0)	3 (12.0)	Ref.	–
Recessive model^2^				
GG	8 (32.0)	12 (48.0)	1.96 (0.62 - 6.19)	0.248 ^NS, b^
AG+AA	17 (68.0)	13 (52.0)	Ref.	–
Alleles [n (%)]				
G	29 (58.0)	34 (68.0)	1.54 (0.68 - 3.49)	0.300 ^NS, b^
A	21 (42.0)	16 (32.0)	Ref.	–

^a^, Fisher’s Exact test;^ b^, Pearson Chi-Square test; %, percent of genotype or allele within group; NS, Non significant at P-value ≥ 0.05; ^1^, Dominant model, (homozygous type + hybrid type)vs. wild type; ^2^, Recessive model,homozygous vs. (hybrid type+ wild type)

**Figure 1:**
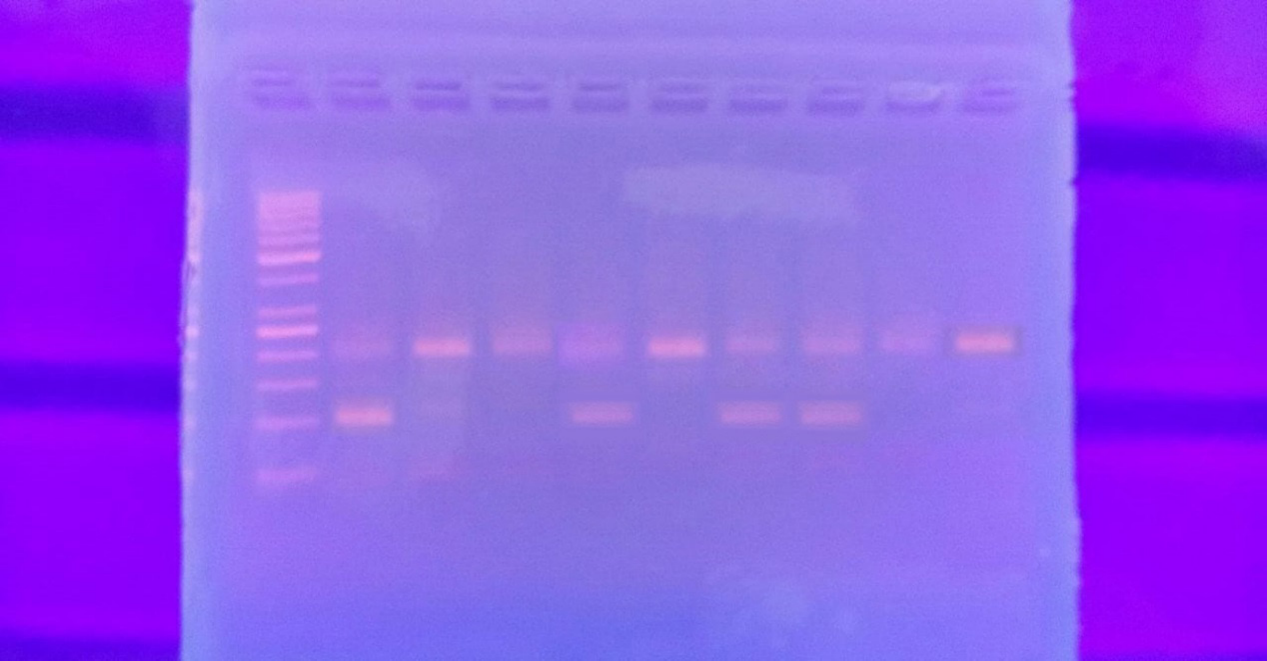
Showing Agarose Gel Electrophoresis after Digestion UsingAlu I Restriction Enzyme for Detection ofEGF 61A > G Polymorphism(rs4444903). Lane 1 50-bp DNA ladder, lane 2, 5, 7 & 8A/G heterozygous (91, 102 and 193 bp bands), lane 3, 4, 6, 9 & 10G/G homozygous (193 bp bands). N.B. 15, 34 bp bands were short and not detected

**Table 5:** Statistical Analysis of Biochemical and Clinical Parametersregarding Genotypes Ofegf Polymorphism (61 A/G) in Resectable HCC Patients

Parameters	EGF Polymorphism (61 A/G)			Significance test	Pairwise comparisons*
	GG (n=8)	AG (n=13)	AA (n=4)		
Age (year)				*χ* ^2^= 3.94	–
Median (IQR)	55.50 (8.50)	61.00 (14.00)	47.50 (17.75)	*P*- value	
Range (min-max)	50.00 - 60.00	49.00 - 71.00	43.00 - 65.00	= 0.139^NS, a^	
Gender [n (%)]				*χ* ^2^ = 0.77	–
Male	7 (87.5)	11 (84.6)	3 (75.0)	*P*- value	
Female	1 (12.5)	2 (15.4)	1 (25.0)	= 1.000 ^NS, b^	
EGF in tumor (T)				F= 4.74	*P*_1_ = 0.032^S^
Mean ±SD	200.00 ± 28.78	162.31 ± 30.86	152.50 ± 35.00	*P*- value	*P*_2_ = 0.050^NS^
Range (min-max)	160.00 - 250.00	110.00 - 210.00	110.00 - 190.00	=0.019^S, c^	*P*_3_ =0.844^NS^
EGF in cirrhotic tissue (C)				F= 0.478	
Mean ±SD	162.50 ± 53.12	138.46 ± 53.52	140.00 ± 73.48	=0.626^NS, c^	
Range (min-max)	80.00 - 220.00	60.00 - 220.00	40.00 - 200.00		
Foci size				*χ* ^2^= 4.40	–
Median (IQR)	4.00 (1.13)	3.00 (1.00)	3.00 (2.00)	*P*- value	
Range (min-max)	3.00 - 5.00	2.00 - 5.00	2.00 - 4.00	= 0.111^NS, a^	
AFP				*χ* ^2^= 0.67	–
Median (IQR)	49.50 (850.45)	36.00 (34.70)	33.00 (44.00)	*P*- value	
Range (min-max)	5.40 - 1327.00	11.50 - 65.00	32.00 - 90.00	= 0.716^NS, a^	

%, percent within group; IQR, Interquartile range; ^a^, Kruskal-Wallis test; ^b^, Fisher’s exact test;^ c^, ANOVA test; NS, Non significant at P-value ≥ 0.05; S, Significant at P-value < 0.05; *, Multiple pairwise comparisons adjusted by Tukey HSDpost hoc test; *P*_1_, P-value for the difference between genotypes GG vs. AG; *P*_2_, P-value for the difference between genotypes GG vs. AA; *P*_3_, P-value for the difference between genotypes AG vs. AA.

## Discussion

Liver carcinogenesis is a complex and multi-factorial process, in which many signaling pathways could contribute to malignant transformation. EGF, through epidermal growth factor receptor (EGFR) acts as mitogen stimulating cellular proliferation and differentiation (Modica et al., 2019). Besides, EGF was suggested to contribute to the occurrence of inflammation and HCC (Berasain et al, 2009 andHuang et al, 2014).

*EGF* gene is 110 kb in length. It contains 24 exons, and is located on human chromosome 4q25. A single nucleotide polymorphism* (SNP)61A>G* located in the 5′ untranslated region influences the expression levels of EGF, where G/G genotype is associated with elevated EGF expression(Wu et al., 2013).

Our present study showed thatA allele was more prevalent in the control group (62%). While, G allele was significantly dominant in HCC patients 63% in HCC patients compared to 42% in cirrhoticpatients. G allele showed significantly high risk for HCC compared to cirrhosis (95% CI) 2.35 (P =0.015). 

These results proved that the G allele may have the risk of hepatocarcinogenesis, while A may be theprotective allele. These results were similar to previous reports (Abu Dayyehet al., 2011- Sun et al., 2015).

Tanabe et al., (2008) demonstrated that the half- life of mRNA transcripts from the G allele was significantly longer than that from A allele. They concluded that the increased stability of transcribed mRNA could explain theincreased risk with G allele. Similar results were reported by Suenaga et al, 2013. 

However, a studyconducted by Qi et al, 2009conflicted our results. Theyfailed to find a significant association between EGF61A/G SNP and risk of HCC(Qi et al, 2009). In addition, a study directed by Gholizadehet al, 2017 on chronic HCV infected Iranian patientsshowed that frequency of the EGF 61A allele in HCC patients was significantly higher than the healthy controls (P value = 0.04).They proposed that the increased risk of HCC with different genotypes might be dependent on the population.

Regarding EGF protein expression in HCC tissue, our present study showed that there was higher concentration of EGF in the tumor tissue (T) (200.00 ± 28.78) in patients with GG genotype compared to AG genotype (162.31 ± 30.86) and AA genotype (152.50 ± 35.00), p= 0.019. This means that functional polymorphism in the *EGF* gene can modifyits protein production.

These results matched withLi et al., (2010)immunohistochemical results of HCC liver tissue. They showed that samples with the GG genotype expressed EGF protein more than those with the AG genotype. In addition, a study conducted by Liu et al., (2018) demonstrated that the expression of EGF in HCCs was significantly higher compared with that in normal tissues, which indicates that EGF is highly expressed in HCC microenvironment. Furthermore, higher level of EGF was significantly associated with higher grade, which suggest that EGF may stimulate progression of HCCs.

To conclude, in the present study,* EGF* gene polymorphism 61*G was associated with increased HCC risk(patients with G/G genotype having more risk than A/G and AA). This is confirmed byincreased EGF expression in tumor tissue of G/G genotype. This could increase the risk of HCC in cirrhotic HCV Egyptian patients. Thus, patients carrying the risk alleles should be closely followed up for early diagnosis and better outcome of treatment.
